# Usefulness of Routine Terminal Ileoscopy and Biopsy during Colonoscopy in a Tropical Setting: A Retrospective Record-Based Study

**DOI:** 10.1155/2014/343849

**Published:** 2014-01-16

**Authors:** Hasitha Srimal Wijewantha, Arjuna Priyadarsin de Silva, Madunil Anuk Niriella, Nethini Wijesinghe, Prabahvi Waraketiya, Ravindu Sujeewa Kumarasena, Anuradha Supun Dassanayake, Janaki de Silva Hewawisenthi, Hithanadura Janaka de Silva

**Affiliations:** ^1^University Medical Unit, Colombo North Teaching Hospital, 11010 Ragama, Sri Lanka; ^2^Department of Medicine, Faculty of Medicine, P.O. Box 06, Thalagolla Road, 11010 Ragama, Sri Lanka; ^3^Department of Pharmacology, Faculty of Medicine, University of Kelaniya, 11010 Ragama, Sri Lanka; ^4^Department of Pathology, Faculty of Medicine, University of Kelaniya, 11010 Ragama, Sri Lanka

## Abstract

*Introduction*. Available evidence for routine terminal ileoscopy during colonoscopy is equivocal. We investigated the place of routine terminal ileoscopy and biopsy during colonoscopy, in a tropical setting. *Materials and Methods*. All consenting adults undergoing colonoscopy had routine TI and biopsy. Patients with right iliac fossa (RIF) pain, diarrhoea, anaemia, suspected inflammatory bowel disease (IBD), and raised inflammatory markers were defined as Group A and all others undergoing colonoscopy as Group B. *Results*. Caecal intubation and TI were achieved in 988/1096 (90.15%) and 832/1096 (75.9%) cases, respectively. 764/832(91.8%) patients were included in final analysis. 81/764 (10.6%) patients had either macroscopic (34/81) or microscopic (47/81) abnormalities of terminal ileum; 20/81 had both. These were CD (28/47), tuberculosis (TB) (6/47), ileitis due to resolving infection (8/47), and drug-induced ileitis (5/47). 27/81 with macroscopically normal ileum had CD (18/27), ileitis due to resolving infection (5/27) and drug-induced ileitis (4/27) on histology. 12/764 (1.57%) patients with macroscopically normal colon had ileal CD (8/12), drug-induced ileitis (2/12), and resolving ileal infection (2/12) on histology. 47/764 (6.15%) patients had ileal pathology that influenced subsequent management. These were significantly higher in Group A (43/555 (8%)) than in Group B (4/209 (1.9%)) (*P* = 0.0048, *χ* ^2^ = 7.968). *Conclusion*. TI and biopsy improve diagnostic yield of colonoscopy in patients with RIF pain, diarrhoea, anaemia, suspected IBD, and raised inflammatory markers.

## 1. Introduction

Terminal ileoscopy (TI) is an integral part of colonoscopy [1]. It confirms completion of colonoscopy. Studies have shown that TI adds only three minutes to colonoscopy procedure time [2]. Furthermore there are no complications in addition to those of colonoscopy [3]. The available evidence for routine TI and biopsy during colonoscopy is equivocal. Some studies have demonstrated a benefit of TI and biopsy in selected patients. These include patients with diarrhoea, right lower quadrant pain, hematochezia, suspected inflammatory bowel disease (IBD), and ileocaecal tuberculosis (TB) [1, 3–6].

Most studies of routine TI during colonoscopy have been performed in Western populations. Only a few studies have been conducted in Asian or other tropical regions [3, 5]. These regions have a different spectrum of gastrointestinal diseases—a relatively low prevalence of Crohn's disease (CD) and higher prevalence of gastrointestinal infections, including TB [5, 7]. Therefore, we investigated the place of routine TI and biopsy during colonoscopy, in a tropical setting, to assess its additional diagnostic yield and impact on patient management.

## 2. Materials and Methods

This was a retrospective study, conducted in the University Endoscopy Unit of the Colombo North Teaching Hospital, Ragama, Sri Lanka. All consenting adult patients (>18 years of age) who underwent colonoscopy from January 2008 to December 2011 were included in the study. All participating patients undergoing colonoscopy had a routine TI and biopsy. Clinical data was obtained from the endoscopy database and patient records and recorded in a preformed data extraction form. Details of the histopathological diagnoses were obtained from the database of the Department of Pathology, Faculty of Medicine, University of Kelaniya, Ragama, Sri Lanka, where all biopsy specimens were processed. The terminal ileum was considered macroscopically abnormal when ulcers, strictures or evidence of inflammation had been reported by the endoscopist. Ileum was biopsied in all patients in whom ileoscopy was performed. Biopsy specimens were taken from visible lesions or, in cases of macroscopically normal ileum, one from each quadrant of the terminal ileum at least 5 cm from ileocaecal valve, using multibite biopsy forceps.

We considered patients with right iliac fossa (RIF) pain, diarrhoea, anaemia, suspected IBD, and raised inflammatory markers as likely to have a higher frequency of ileal abnormalities (“definite indication” for TI and biopsy: Group A) than patients undergoing colonoscopy for other indications (“no definite indication” for TI and biopsy: Group B). The macroscopic and microscopic findings of the terminal ileum were also compared between these two groups. Chi-square test was used for the comparison between the two groups. A *P* value of <0.05 was taken as statistically significant.

Ethical clearance for the study was obtained from the Ethical Review Committee of the Faculty of Medicine, University of Kelaniya Ragama, Sri Lanka.

## 3. Results 

**Figure 1:**
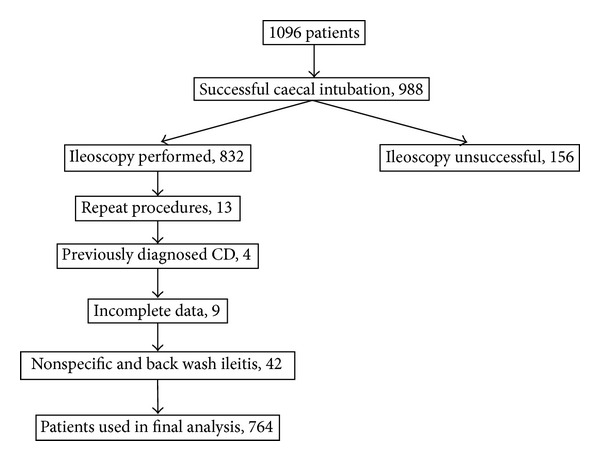
Patient selection.

A total of 1096 colonoscopies were performed per protocol during the study period (see Figure 1). Successful caecal intubation was achieved in 988/1096 (90.15%) and terminal ileum was intubated in during the study period, 4 patients with previously diagnosed CD and 42 patients with nonspecific ileitis or backwash ileitis in ulcerative colitis (UC) on histology were excluded. Therefore, only 764/832 patients were included in the final analysis.

**Figure 2:**
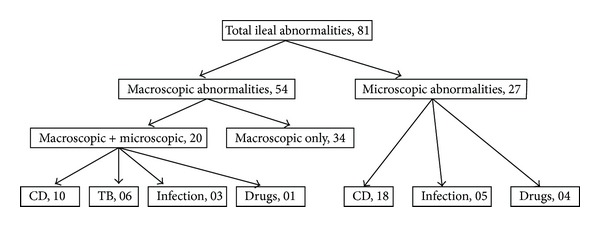
Macroscopic and microscopic abnormalities of the ileum.

Indications for colonoscopy in the patients with successful TI are given in Table 1 81/764 (10.6%) patients had either macroscopic (34/81) or microscopic (47/81) abnormalities of the terminal ileum while 20/81 patients had both (see Figure 2). Overall 47/764 (6.15%) patients were diagnosed to have significant ileal pathology that changed the management of the patient or provided clinically useful information: CD (28/47), TB (6/47), ileitis due to resolving infection (8/47) or drug-induced ileitis (5/47; subsequently diagnosed as probable nonsteroidal anti-inflammatory drug-induced).

Macroscopic abnormalities of the terminal ileum described were ulcers, strictures or evidence of inflammation. Microscopic abnormalities described were CD, ileal TB, resolving infective ileitis and drug-induced ileitis. 27/81 patients had microscopic ileal abnormalities with normal macroscopy. Their histological diagnoses were CD (18/27), ileitis due to resolving infection (5/27) and drug-induced ileitis (4/27). Patients with macroscopic abnormalities of the terminal ileum had significantly higher frequency of histopathological abnormalities (CD, TB, drug-induced ileitis, and infective ileitis) when compared with the patients with macroscopically normal ileum (*P* < 0.0001, *χ* ^2^ = 161.57) (see Table 2). There were 34 patients with ileal abnormalities on endoscopy with normal histopathology on biopsy. On ileoscopy, 21/34 of them had been described as having mild ileal inflammation or few erosions and other 13/34 patients had been found to have few aphthous ulcers. 630/764 (82.46%) patients had no macroscopic mucosal abnormality of the colon. 12/630 (1.9%) patients with endoscopically normal colon were diagnosed to have CD (8/12), drug-induced ileitis (2/12) and resolving infection (2/12) on TI and biopsy.

There were 555/764 (72.64%) patients in Group A and 209/764 (27.35%) patients in Group B. The groups were comparable for age and sex distribution (Group A and B: mean age (SD) 48.8 (16.5) and 49.9 (15.4); Student *t*-test *P* = 0.4; *M : F* ratio 1 : 1.08 and 1 : 1.05; *P* = 0.92, *χ* ^2^ = 0.008). Macroscopic abnormalities of the terminal ileum were commoner among patients who had a definite indication for ileoscopy (Group A—29/555) when compared patients who did not (Group B—5/209) though it was not statistically significant (*P* = 0.1347, *χ* ^2^ = 2.238). Histopathological abnormalities of the terminal ileum (that changed the management of the patient or provided clinically useful information) were significantly higher among patients of Group A [43/555] than in Group B [4/209] (*P* = 0.0048, *χ* ^2^ = 7.968).

## 4. Discussion

We found that 10.6% of our patients had either macroscopic or microscopic abnormalities in the terminal ileum. This is a higher figure compared to studies conducted in Western countries [2, 4, 8]. Western studies have shown a 2% to 7.2% diagnostic yield when routine TI was performed in unselected patients [5]. In one study, the diagnostic yield of routine TI was as low as 0.3% [8]. This study had been carried out on a selected population: asymptomatic patients undergoing screening colonoscopy [8]. In most such studies, the ileum had been biopsied only when there was an endoscopically detectable abnormality [5, 8] and Crohn's ileitis was the diagnosis made in most cases. In contrast, nearly all of our patients were symptomatic or had an indication for colonoscopy (Table 1). Furthermore, ileal biopsy was carried out irrespective of the endoscopic appearance of the terminal ileum. We did not observe any major complications related to TI and biopsy.

Patients with known ileal CD were not included in the study as they were expected to have ileal abnormalities. Patients who had nonspecific ileitis or back wash ileitis due to UC on histopathology were excluded from final analysis as they did not affect the management of the patients. Only the patients with CD, TB, drug-induced ileitis and ileitis due to resolving infection were considered as significant abnormalities for purposes of our analysis as their importance was unequivocal. Five patients were diagnosed to have drug-induced ileitis. The diagnosis was made in retrospect after histopathology in patients who underwent colonoscopy for other complaints. Drug history had not been recorded at the time of ileoscopy. However, at the subsequent clinic visit, these patients admitted to have taken nonsteroidal anti-inflammatory drugs during or around the time of their symptoms.

The yield of ileoscopy would depend on the clinical presentation of the patient. In our study we considered that it is likely that patients with RIF pain, diarrhoea, anaemia, suspected IBD and raised inflammatory markers would have a higher frequency of ileal abnormalities than patients undergoing colonoscopy for other indications. The rationale for this hypothesis was that it would include patients with conditions that would commonly give rise to ileal abnormalities such as CD, TB, and other types of ileitis which would influence management decisions. We have clearly shown that ileal abnormalities are significantly higher among patients with such indications (Group A) than those without such indications (Group B) confirming the findings of previous studies [1, 3–6].

There were 27/764 (3.53%) patients with significant ileal abnormalities on histology despite having an endoscopically normal terminal ileum. Among them there were 18 patients with CD. 82.46% patients did not have a mucosal abnormality of the colon on endoscopy. However, 1.9% of these patients were found to have a significant ileal abnormality (CD, ileitis due to resolving infection, and drug-induced ileitis) on TI and biopsy. The diagnosis would not have been made in these patients had TI and biopsy not been performed. A study from India has also shown a high diagnostic yield of TI, and in that study 14% (8/57) with ileal abnormalities had a normal colonoscopy or barium enema [3].

Cases where the macroscopic appearance of the ileum was reported as abnormal but histology was normal are difficult to explain. These may reflect the subjective nature of some endoscopy reports or may have been due to a policy of “biopsy when uncertain” when the endoscopist was not confident regarding a macroscopic appearance.

28/764 (3.6%) patients were diagnosed to have CD on ileal biopsy in this study. All patients with histology suggestive of CD had a negative TB-PCR and TB culture. A hospital based survey carried out in two districts of Sri Lanka found the prevalence of CD to be 1.2/100,000 population [7], much lower than in Western series. However, the present study was conducted in a tertiary referral center with a special interest in IBD. The high frequency of CD among patients undergoing colonoscopy in our unit is likely to be due to a referral bias. Although this study was conducted in a tropical setting, we did not observe a high rate of infectious diseases. Only 14/764 (1.8%) of cases had evidence of TB or ileitis related to a resolving infection. Even after accounting for a referral bias, the frequency of ileal infections appears to be low.

## 5. Conclusion

We believe that TI and biopsy should be an integral part of colonoscopy in patients with RIF pain, diarrhoea, anaemia, suspected IBD or raised inflammatory markers. It improves the diagnostic yield of colonoscopy and influences management by giving additional information in this group of patients, sometimes even when the macroscopic appearances of the colon and the terminal ileum are normal. Furthermore, TI and biopsy can be performed relatively easily and without additional risk of adverse effects. However, we did not find strong enough evidence to recommend its routine use in all cases, as the diagnostic yield in those without “definite indications” for an ileoscopy was low. The additional cost of routine ileal histology may be a further drawback in the latter group.

## Figures and Tables

**Table 1:** Indications for colonoscopy.

Indication for colonoscopy	Number of patients	Percentage (%)
Group A		
RIF pain	121	15.83
diarrhoea	224	29.31
Anaemia	75	9.81
Suspected IBD	81	10.6
RIF pain and diarrhoea	14	1.83
Anaemia and diarrhoea	6	0.78
Raised inflammatory markers	6	0.78
Bleeding PR and RIF pain	8	1.04
Bleeding PR and Diarrhoea	20	2.61
Subtotal	**555**	**72.64**

Group B		
Polyps	7	0.91
IBS	29	3.79
Loss of weight	17	2.22
LIF pain	22	2.87
Constipation	60	7.85
Bleeding PR	45	5.89
Other	29	3.79
Subtotal	**209**	**27.36**

Total	**764**	**100.00**

**Table 2:** Histopathological abnormalities of the ileum in Group A and Group B.

Histopathological diagnosis	Group A (*n* = 555)	Group B (*n* = 209)	Total (*n* = 764)	Percentage (%)
Crohns disease	24	4	28	59.57
Tuberculosis	6	0	6	12.76
Ileitis-resolving infection	8	0	8	17.02
Drug induced ileitis	5	0	5	10.64

Total	43	4	47	100.00
